# A Multi-Protocol IoT Platform Based on Open-Source Frameworks

**DOI:** 10.3390/s19194217

**Published:** 2019-09-28

**Authors:** Charilaos Akasiadis, Vassilis Pitsilis, Constantine D. Spyropoulos

**Affiliations:** 1 Software and Knowledge Engineering Laboratory, Institute of Informatics and Telecommunications, NCSR ’Demokritos’, Aghia Paraskevi 15341, Greece; costass@iit.demokritos.gr; 2 Division of Applied Technologies, NCSR ’Demokritos’, Aghia Paraskevi 15341, Greece; vpitsilis@dat.demokritos.gr

**Keywords:** IoT platform, open-source frameworks, interoperability, multiple application layer protocols, IoT ecosystem

## Abstract

Internet of Things (IoT) technologies have evolved rapidly during the last decade, and many architecture types have been proposed for distributed and interconnected systems. However, most systems are implemented following fragmented approaches for specific application domains, introducing difficulties in providing unified solutions. However, the unification of solutions is an important feature from an IoT perspective. In this paper, we present an IoT platform that supports multiple application layer communication protocols (Representational State Transfer (REST)/HyperText Transfer Protocol (HTTP), Message Queuing Telemetry Transport (MQTT), Advanced Message Queuing Protocol (AMQP), Constrained Application Protocol (CoAP), and Websockets) and that is composed of open-source frameworks (RabbitMQ, Ponte, OM2M, and RDF4J). We have explored a back-end system that interoperates with the various frameworks and offers a single approach for user-access control on IoT data streams and micro-services. The proposed platform is evaluated using its containerized version, being easily deployable on the vast majority of modern computing infrastructures. Its design promotes service reusability and follows a marketplace architecture, so that the creation of interoperable IoT ecosystems with active contributors is enabled. All the platform’s features are analyzed, and we discuss the results of experiments, with the multiple communication protocols being tested when used interchangeably for transferring data. Developing unified solutions using such a platform is of interest to users and developers as they can test and evaluate local instances or even complex applications composed of their own IoT resources before releasing a production version to the marketplace.

## 1. Introduction

The rapid growth in computer communication technology and the application of networked system solutions into every area of our daily lives as well as the increasing number of devices that use such networks create many prospects for subsystem interconnections, provisioning a new generation of cyber-physical services widely referred to as the Internet Things (IoT) [1]. Such services can be incorporated by solutions for a variety of use cases, e.g., related to health, energy, industry, etc., so to improve people’s routines and businesses [2,3]. The unification of available and heterogeneous IoT resources—i.e., devices and software services—into a pool of publicly available solutions is highly desirable, and such a concept would help outspread IoT-enabled applications [4]. At the same time, however, interoperability, connectivity, and scalability issues arise, both at the device and platform levels. These issues disturb the universal adoption of IoT solutions and delay the development of new services [5].

Interoperability, in particular, refers to wide capabilities for interconnecting heterogeneous platforms, systems, and services in order to fully cooperate with each other in every aspect of their operation, e.g., turning on/off, changing configuration, exchanging sensor information, etc. [6,7]. The vision is to create seamless interconnections and to provide an infrastructure for the automatic discoverability, configuration, and execution of complex IoT services and platforms [8,9]. Still, recent work highlights the interoperability requirement, and convergence to specific technologies, e.g., a single standard, is deemed unlikely [10,11]. Considering its importance, the feature of interoperability should be integrated into multiple layers, i.e., data, applications/services, middleware, and networking, and into the device level [4,12]. Moreover, interoperable services should also be reusable even in different application domains than those originally designed for [13]. Reusability, which also includes service reconfiguration capabilities, is imperative for deploying large-scale, sustainable, and self-organizing IoT ecosystems for many application areas, such as ambient assisted living, smart energy, smart transport, and so on [5].

Now, a key layer of the IoT stack regarding interoperability is the so-called middleware. This layer is responsible for providing seamless interconnections between heterogeneous resources and is also responsible for the exchange of data and messages across various domains and interfaces [14]. Importantly, IoT middleware must be easy to use and should provide means for services interconnection, data analytics, context awareness, and resource management and control via graphical user interfaces [15]. Although still an active area of research, certain approaches currently exist that offer connectivity, flexible service composition, and discovery, as well as privacy and security [16]. Typically, middleware approaches implement one or more Application Layer Protocols (ALPs) [17]. ALPs undertake the communication requirements among IoT services and devices and are used to control the flow of data, i.e., sensor measurements, commands, etc. Currently, quite a few ALPs exist, often competing with one another, with none so far managing to conquer the global market and to be characterized as the most dominating in the IoT sector. Differentiating factors of ALPs are the serialization formats; the communication patterns, e.g., publish/subscribe or request/response; and the available quality of service [18]. However, supporting multiple protocols is a key requirement for allowing cross-layer communications and for enabling the creation of virtual brokerage-based systems for ambient intelligence and context-aware applications [19].

This paper mainly focuses on the interoperability features of IoT ecosystems. We present an IoT platform that use open-source middleware frameworks and that integrates them into a single operable instance. The proposed approach builds upon SYNAISTHISI [20], a cloud-based and scalable IoT application-enabler platform which has been expanded to support multiple ALPs, i.e., Message Queuing Telemetry Transport (MQTT), Representational State Transfer (REST)/HyperText Transfer Protocol (HTTP), Websockets, Constrained Application Protocol (CoAP), and Advanced Message Queuing Protocol (AMQP), as well as semantic descriptions, user access control, and IoT resource management. The platform is available in the form of interconnected dockerized containers that serve different requirements of the IoT stack. In particular, except for bridging information over multiple ALPs by incorporating RabbitMQ and Ponte open-source brokers, the solution that we examine supports the authentication and authorization procedures regarding access and ownership of data streams and cloud-based micro-services and the graphical user interfaces that are used for the monitoring and management of the services execution. To support the oneM2M technical standard, the open-source OM2M framework is included and interfaced with the other components of the platform, as well. By offering multiple interconnected protocol interfaces to the users, existing services based on different cases can seamlessly interconnect with each other and can form complex and reconfigurable cyber-physical applications. It is in our intentions to evaluate the proposed platform with respect to its performance when multiple ALP protocols are used. This is done by conducting simulated experiments and stress tests.

The capability to unify fragmented IoT applications is of high interest to teams of developers that work for both business and research domains. To this end, it is important to allow large-scale service *reusability* and to also monitor and track usage of resources from the end users. To achieve this, we follow a marketplace-oriented architecture that incorporates semantic descriptions to enable service discovery. The platform is available for local instance deployments; thus, developers can test and evaluate their applications before offering them for further use, which aims to create a large pool of IoT resources, active developers, and end users towards the realization of an open large-scale IoT ecosystem.

The rest of this paper is structured as follows. In Section 2, we present some of the related notions and the research that has been performed so far regarding ALPs and the existing IoT platform architecture approaches. Then, in Section 3, we present the internal architecture of the platform’s containerized version and the used database model. Section 4 includes a discussion from an IoT marketplace perspective, examples of the process required to design new IoT-enabled services, as well as the design principles for complex IoT service approaches that can be used in ambient intelligence use cases. In Section 5, we present illustrative results from simulation experiments, and finally, we conclude in Section 6.

## 2. Background and Motivation

As stressed earlier, the vast majority of the currently available interconnected systems approaches is implemented for specific and fragmented application domains, lacking the capability to unify solutions, which is an important feature from an IoT perspective. This deficiency does not allow the manipulation of reusability and reconfigurability properties of systems and services that is a significant requirement for the IoT case [3,11].

One way for delivering such capabilities is to use semantics. Many ontologies and data models have been proposed regarding the IoT domain for devices, data, and IoT resources in general, e.g., the IoT-Lite [21], which is tailored to sensor networks; the SAREF (Smart Appliances REFerence) ontology [22], which describes smart appliances and was defined by collaborating with the industrial sector; the Ontology for Wearables Data Interoperability [23], which can be used for ambient assisted living applications; and IoT-A [24]. Such semantic descriptions can help with the discoverability of devices and services and with their automated reconfigurability and management. A good example of how semantics can be incorporated into IoT solutions for real-time stream processing is the CityPulse project [25]. This work highlights the benefits of using ontology models to describe the nature of measurements in each stream as well as to assess the quality of features and information that are exchanged. Still, ontologies and semantics are not always incorporated in the available IoT platforms.

A number of surveys already exist regarding IoT platforms and the comparison of their capabilities from many aspects. For example, Reference [26] evaluates platforms according to various application domains, such as application development, device management, analytics, research, etc. An existing open-source platform that is also offered alongside commercial services is Mainflux [27]. This platform incorporates multiple application layer protocols, applies user access controls on data streams and devices, and comes in the form of dockerized image with the option to include Grafana and InfluxDB for time-series data storage and management. However, Mainflux does not focus on service reusability and reconfigurability as SYNAISTHISI does [20]. Also, the infrastructure for semantic descriptions of services and data streams is not defined, and more attention is given on the back end of IoT platforms infrastructure. This way the platform diverges from a marketplace-oriented approach.

Extensive comparisons of already available approaches, either open-source or proprietary, are given in References [15,16,26,28]. Examples of the most known platforms are EVRYTHNG, Kaa, SmartWorks, ThingSpeak, ThingWorx, Ubidots, and Xively (https://evrythng.com, https://www.kaaproject.org, https://www.altairsmartworks.com/, https://thingspeak.com, https://www.ptc.com/en/products/iiot/thingworx-platform, https://ubidots.com/, https://xively.com/). In their vast majority, such solutions are supported by specific organizations and companies that nevertheless aim to engage as much user base as possible with the risk to create further fragmentations on an ecosystem-wide level, additionally to the application domain level, where such issues currently exist. Moreover, they are offered as complete products and attention is not paid on open research dimensions. This fact can mislead users to believe that these solutions are mature enough for real-world deployments, while many features still need to be researched and tested, for example, privacy and security, sustainability, etc. [29,30].

Another debate that exists in this field regards the choice between proprietary software or open-source approaches. The merits from adopting open-source solutions are quite a few, e.g., some common issues are already solved by such software; the cost to obtain and integrate is quite lower; often, specific standards are implemented; etc. [17]. Yet, risks are also present, such as the possibility of product discontinuation; unclear market domination of one solution over another; and frequently, the management of open-source development teams being a challenging task. Nevertheless, many large software corporations have made the turn towards adopting or contributing open-source solutions.

The IoT platform gap analysis presented in Reference [31] compares a number of existing IoT platforms and highlights specific recommendations for delivering contemporary IoT solutions that match current ecosystem needs. Specifically, these include the ability to support heterogeneous devices; to effectively manage data ownership, processing, and sharing; to provide support for developers; and to incentivize the creation of ecosystems and marketplaces.

### 2.1. IoT Marketplaces

A few years earlier, the openIoT research project [32] defined an IoT service framework, which was designed to address the massive IoT market needs and to establish a global IoT ecosystem. Stakeholders are divided into six main categories, according to whether they provide, develop, operate, or use software, devices, and infrastructure resources. Each category is then characterized by its “gives and takes”, clarifying potential revenue streams and applicable business models.

Later, towards an interoperable IoT infrastructure, the BigIoT platform was created, which offers an open Application Programming Interface (API) for IoT resource interconnections [33]. BigIoT incorporates a marketplace approach, where resources can be exposed, rated, and shared with others via a web portal. On a similar track, INTERIoT [4] defines different IoT stack layers, of which adoption can significantly help in platform federations, IoT resource sharing, and the creation of large-scale ecosystems in general. Both these approaches, along with symbIoTe, bIoTope, AGILE, Vicinity, and TagItSmart, are part of the European Platforms Initiative (https://iot-epi.eu), an organization that aims to deliver sustainable IoT solutions with focuses on platform development, interoperability, and information sharing on the various technology stack layers. Such actions are very effective for promoting the broader vision, for establishing guidelines, and for delivering prototypes; however, quite often, offered services are no longer supported by the time a research project is completed and funding stops.

The IoT platform survey presented in Reference [34] highlights the recent trend of having end users develop or customize their own IoT applications and products. To meet this market requirement, new, lower-level, open hardware and software platforms have been developed and are currently available for acquisition by consumers and end users, e.g., IoTivity, LittleBits, and WeIO (https://iotivity.org/, https://littlebits.com/, http://we-io.net/hardware/).

Facing these facts, a platform should use an online marketplace approach, where end users not only may select existing services to reuse and integrate into their applications but also may offer their own to be used by third parties according to a preselected sharing policy. However, the marketplace architecture is not enough to promote interoperability, and a variety of other technical issues have to also be taken into account, e.g., protocols for service communication, federation with other platforms, and means for engaging the user base.

### 2.2. Application Layer Protocols

The term *application layer protocol* refers to a protocol that manages data and information exchanged between devices and the final software applications [35]. A device can either be a single sensor; a gateway that integrates a larger number of sensors; or even actuating devices, such as motors, lighting equipment, etc. However, in the present state of IoTs, multiple application layer protocol specifications are available for use and none of them has managed to become the common standard used in such systems and applications. Examples of protocol specifications include MQTT, CoAP, Websockets, AMQP, REST, etc. The available protocols often follow one of the two most common architecture approaches, the *request/response* or the *publish/subscribe*, each having different advantages and drawbacks [36]. Also, each protocol possesses different features, i.e., quality of service options, security mechanisms, etc. In particular, Reference [37] presents a performance analysis paradigm between MQTT and CoAP in scenarios where erroneous transmissions exist. The comparison shows that there are differences between these protocols under different circumstances, e.g., high traffic, packet loss probability, low latency required, etc. The Industrial IoT Connectivity Framework published by the Industrial Internet Consortium defines a stack and a reference architecture to help practitioners decide on the suitability of a connectivity-related solution [12]. Our focus in this article is on the transport and framework layer of the connectivity stack presented therein, where messages are exchanged and structured data are generated and manipulated. These layers constitute the technical and syntactical aspects of interoperability and should allow the integration of various ALPs by using protocol translation and data-manipulation modules. Core function categories of the framework layer that we also integrate in our approach are *security*, e.g., authentication and authorization and quality of service; *data type and resource models*, e.g., semantic descriptions; and *data-access protocols*, e.g., communications architecture, data discovery, application programming interfaces, etc.

In Reference [38], the authors present a system architecture for benchmarking different IoT middleware. Three distinct and commonly used protocols are tested and compared with respect to publish and subscribe times, generated network traffic, and success rates. An extensive performance comparison of ALPs used in IoT applications is given in Reference [18]. This work examines the characteristics of six different protocols and assesses their performance with respect to latency, data throughput, energy consumption, security, and programmer’s choice. Importantly, the authors also evaluate the suitability of each protocol for incorporation in various levels of the technology stack, i.e., IoT, Fog, and Cloud. Results show that MQTT and REST/HTTP are currently the most widely accepted and supported protocols; however, CoAP might as well establish itself as an IoT messaging standard in the future.

Reference [39] provides a low-level comparison between MQTT and MODBUS, and promotes their combination for usage in industrial IoT applications, where interoperability is an imperative requirement. In Reference [40], the ATLAS IoT communication framework is presented, which supports seamless communication between devices that recognize CoAP, REST/HTTP, and MQTT protocols. This approach is focused on a protocol translator that can be deployed either on a cloud infrastructure or on the IoT device itself. The approach is also compared to Ponte, an open-source framework that we also examine, and experiments illustrate reduced energy consumption when using REST HTTP and CoAP ALPs. In what follows, we describe the architecture of a platform compatible with the desired characteristics that we have discussed and that promotes IoT solutions unification. We present the various open-source components that the platform is composed of and the configurations required for their interconnection. Our objective in this work is not to advertise another IoT platform but to explore and investigate existing open-source frameworks that can be used to compile a custom platform that adheres to contemporary standards.

## 3. Platform Architecture

Taking into account the motivation of the previous section, here, we accumulate a list of desired characteristics and combine existing open-source components that form an operable IoT platform. In particular, a contemporary IoT platform should provide the following features: (a) seamless interconnections between services and datastreams; (b) support for multiple application layer protocols; (c) ability to be deployed on a large scale; (d) ability to monitor and control ownership and sharing of services and datastreams; (e) support for ontologies and semantic descriptions; (f) promotion of service reusability in electronic marketplaces; and (g) guarantee of secure and private data access and communication for services.

**Figure 1:**
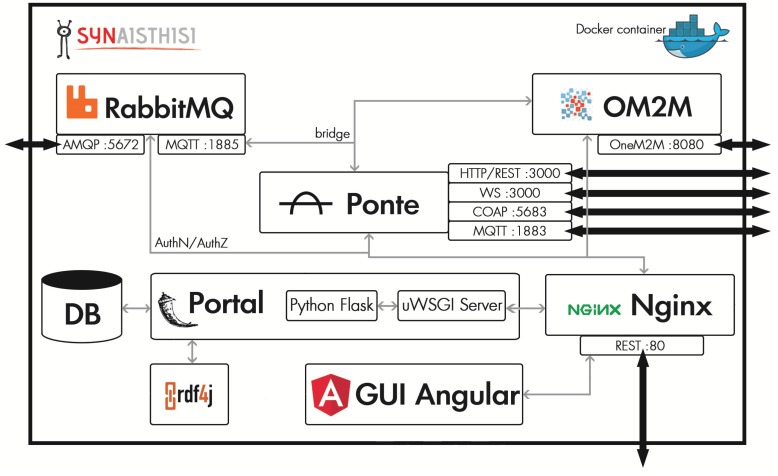
Platform components

To this end, the examined platform solution provides interfaces that support five of the most commonly used ALPs, i.e., MQTT, AMQP, Websockets, CoAP, and REST HTTP. It is based on open-source components that are effectively interconnected and that provide support for (i) the oneM2M common service layer; (ii) semantic descriptions that can be used for service discoverability and reasoning; and (iii) authentication/authorization (AuthN/AuthZ) procedures for services and data-stream access and sharing. To integrate these functionalities, we set up a SYNAISTHISI platform instance as a dockerized container and incorporate open-source message brokers that interconnect and cross-translate the five ALPs. Additionally, we include the OM2M framework that supports the oneM2M common service layer specification and the RDF4J triplestore for storing and processing semantic data.The modules and internal organization of the platform’s components is depicted in Figure 1. More details regarding our choice on the various submodules are given below.

### 3.1. Docker

Docker uses *containers* to run sandboxed instances of processes [41]. These containers can be executed on top of existing operating systems and are isolated from each other as well as from other components of the hosting operating system. This approach allows dynamic use of resources, meaning that, if a process is not executed, then the Central Processing Unit (CPU) can be used for other purposes as well as for a faster start time. Instead of powering on and off a computer (virtual machine case), we just initiate or “kill” an application (container case). Virtualization is performed on the operating system level in contrast to virtual machines where the virtualization is on the hardware level.

Docker has a wide area of applications in software and web-engineering solutions and has been used for reproducing results of research, a problem that has been troubling the scientific community for many years [42]. One of the merits for adopting Docker-oriented deployments is that all the necessary dependencies of the system, i.e., libraries, configurations, data, and source code, are shipped together as a single image. The procedure for setting up the system is described in the form of a script that contains the instructions required for the setup on possibly different operating systems than the one that the container is hosted on. This script can then be easily shared with the community, and its execution will yield the same results each time.

In our approach, we offer a containerized version of the platform to the users, so that they can deploy it in “local” instances either to test their IoT solutions or to deploy them in constrained environments. It is important to note that the Docker engine, that is, the infrastructure required to run a SYNAISTHISI container, has available versions for all types of modern operating systems (e.g., MS Windows, MacOSX, Ubuntu, etc.).

### 3.2. Ponte

Ponte (https://www.eclipse.org/ponte/) is an open-source project of the Eclipse Foundation that has attracted the attention of the developer and research communities [40,43,44,45]. This framework bridges various ALPs, i.e., MQTT, MQTT over Websockets, REST/HTTP, and CoAP, allowing micro-services implementing different protocols to communicate with one another. This is achieved by orchestrating different message brokers based on well-known examples, such as Mosquitto and RabbitMQ. Ponte is implemented in Node.js and allows the incorporation of third-party authentication and authorization services for access to data streams, making it a perfect candidate to serve as the main cross-translator of ALPs in the platform approach that we propose. Also, it is possible to use external message brokers other than those that are shipped with the framework, and the same holds for databases for persistence.

### 3.3. RabbitMQ

RabbitMQ (https://www.rabbitmq.com/) is one of the most popular open-source message brokers, and similarly to Ponte, it also supports multiple messaging protocols, i.e., AMQP 0-9-1, AMQP 1.0, STOMP, MQTT, and HTTP [46,47,48]. Apart from the additional protocol support, another desired feature of RabbitMQ from an IoT platform perspective is that it can be configured as a cluster of distributed brokers and, thus, can constitute a highly available solution. In our platform, RabbitMQ’s MQTT binding serves as an isolated bridge between RabbitMQ, Ponte, and OM2M. Access rights, as they are defined in the back-end server, are replicated to the framework’s default module so that access based on AMQP and STOMP protocols is effectively controlled with the same user credentials.

### 3.4. OM2M

The Eclipse OM2M project (https://www.eclipse.org/om2m/) is based on the European Telecommunications Standards Institute (ETSI) set of specifications, which define a common Machine-to-Machine (M2M) service platform [38,49,50]. OM2M is based on an OSGi layer [51], allowing the integration of other plugins, as well as additional ALPs and device management mechanisms. By default, OM2M defines a RESTful API that is used to exchange Extensible Markup Language (XML) data even when highly unreliable networking is used. The modules of this framework are organized in a service capability layer to provide many desired features, such as authentication, resource discoverability, synchronous and asynchronous communications, etc. [52]. Similarly to the RabbitMQ case, in order to interconnect OM2M with our platform, we used OM2M MQTT binding, which is bridged with the main MQTT broker of the container and therefore translated to every other supported protocol as well (see Figure 1).

### 3.5. RDF4J

The Eclipse RDF4J (http://rdf4j.org/) open-source framework is implemented in Java, and it is used to process Resource Description Framework (RDF) data, including their parsing, storing, querying, and inferencing. In our case, it has been selected as the host of ontologies that we incorporate for semantically annotating IoT resources. Apart from a basic graphical user interface, RDF4J exposes an API to programmatically connect and extract or update/enrich information in the platform’s RDF repository, which is also used by our back-end server to annotate new or to discover existing IoT resources.

### 3.6. Sub-Module Interconnection

In the examined implementation, the main pillar of the dockerized container is an NGINX server. NGINX is a software solution that is configured here to provide a web server with high-performance load balancing (https://www.nginx.com). Both Graphical User Interface (GUI) and AuthN/AuthZ requests are submitted to NGINX and are consequently routed to the relevant sub-modules. The portal and the custom back-end server, apart from providing information regarding AuthN/AuthZ operations and IoT resources content and descriptions to the GUI, are also queried by the message brokers implementing the various ALPs. IoT resources and user information are stored in a database inside the container; however, it can be configured to include any other endpoint, local or remote, or centralized or distributed. The portal also requests information related to the semantic annotations of the IoT resources from the RDF4J triplestore. Regarding messaging, Ponte is configured to use an external MQTT broker in its back end, which in our case is the one provided by RabbitMQ, running on port 1885. This particular broker is also linked to the MQTT binding of OM2M and serves as the bridge that is used for translating messages into the various ALPs. The actual endpoint that clients connect to the MQTT broker is exposed by the Ponte framework on port 1883. Thus, to avoid security concerns, the “bridge” MQTT broker running on 1885 is not exposed to the outside of the containerized instance. More details regarding the components and their interconnection are visualized in Figure 1.

Note that historical data exchanged by services via topics are not stored anywhere on the platform in its current version. To keep track of historical measurements from sensors or processed data from services, the interested party must deploy a specialized service provided for this purpose and also have a storage infrastructure available, e.g., a MongoDB instance installed on premises or on a different cloud server.

### 3.7. Database Model

One of the main goals of the examined platform is to give users the capability to manage and unify micro-services, which can be loosely connected with one another, so as to exchange information and to create ad hoc relationships based on which datastreams may flow from one service to another. Thus, an added value chain of services and information is created that can be incorporated into a plethora of IoT applications, such as smart cities, ambient intelligence (AmI), smart health, etc. In order to ensure (a) that access to the exchanged information is controlled and explicitly granted or denied and (b) that this information is properly grouped and processed, we provide a data model that keeps track of permissions and proper interrelation between all key entities and that is used by the Flask portal.

The proposed data model ensures that all the required entities are represented and that they are interrelated in a way that their unobstructed interconnection is achieved. The main entities of our application are *users*, *topics*, and *services*.

- **Users** represent the physical persons who create services and handle their data. Users should be able to manage their resources, i.e., enable their devices to send and acquaint data and to create and manage services that process such data.
- **Topics** are the communication channels established by the message brokers that enable the publishing and reception of IoT resource input and output, e.g., measurements, states, commands, etc. All users’ devices (e.g., sensors) share their data with other authorized user services or devices by publishing to these communication channels. The adopted permissions control process enables or disables access to these data.
- **Service** entities correspond to an abstract group of communication channels (topics) bundled with an algorithm operating on the data that are received by these channels. Service functionality, i.e., the actual algorithm, is implemented in an interconnected service file, and results are subsequently published to (output) topics where third-party clients (services or devices) can subscribe and make use of such information.

For each of the entities above, there is a corresponding table in the database that holds all related information. For users, registration data is stored, such as the credentials used for identification and authentication, an e-mail address, the user’s role in the platform, and registration and confirmation data. For topics, we store a name and a description, and for services, we store their (unique) names; a description; an owner “id”, i.e., a foreign key to the users table, which indicates who created and has the administrative responsibility for this particular service; the location; the service type (sensor, actuator, or processor); and a field which keeps some ontology information used to classify the service based on a well-established vocabulary. Also, both topics and services have fields related to the sharing policies and pricing in case another user would like to receive access. All these entities are indexed based on a unique (per table) “id” field.

With these descriptions in mind, we can see that user authentication is enabled based on the information stored in user entity (username/email and password, which act as the user credentials). What is equally important is the authorization of users on accessing instances of the other two entities. In particular, we are interested in the capability to answer if specific users are authorized to publish, retrieve, and process information.

In the first level, it is crucial to control users’ permissions to publish information in broker’s communication channels and, then, users’ permissions to retrieve (or subscribe to) this information. This means that, using each topic, every user may (or may not) publish data and may subscribe to receive data published through it. In order to control these permissions and to handle authorization to broker topics in the database, we implement a relationship-enforcing table which is called users_topics_map table. In this table, there is a row per user and topic if and only if the user has any kind of permissions on this topic. Such information is crucial as it is used to provide authentication each time a user (using, e.g., a service or a device) requests access to send (publish) or get (subscribe) information to and from the broker. In fact, the user tries to subscribe or publish to a broker topic and the broker forwards the request to the application portal, which responds (positively or not) according to the value stored in this table for the specific user and topic.

In our platform, IoT services are compiled as (a) a set of input topics (on which they *subscribe* based on their owner permissions on these topics) from which they get data and process them based on the business logic they implement and then publish newly produced data on one more more output topics (on which they can *publish* based on their owner permissions on these topics as explained above) and (b) a service (source code) file which implements the subscriptions and publishing to the broker topics as well as the algorithm which implements the service business logic. The proposed data model keeps the information of which topics are connected, either as input or output topics, to which service. Note that each service should at least have one topic as either input or output. This information is stored in a services_topics_map table, which has the fields service_id(fk), topic_id(fk), input(boolean), and output(boolean). Therefore, for every topic connected to a service, there is a row which indicates the service and the topic and which indicates whether this topic serves as an input or an output for this service. For example, in order to get all topics connected to a service, we must query this table for all rows which have a specific service_id. In reverse, if we want to know, e.g., to which services a specific topic serves as an input, we have to query for all rows which have a specific topic_id and a value of the input field as True.

Now, each service has an owner that is identified by the foreign key owner_id, but each user that requests the permission to use this service obtains a record in the table users_services_map, which then keeps a record for every user and service that is related, i.e., the user is granted permission to use the service. This way, we can keep track of information related to which services users are permitted to use. It is important to note that, when a user requests and subsequently is granted permissions to use an existing service, this implies that the specific user is granted subscribe permissions to this service’s *output topics* (namely, this user can obtain the result produced by this service) and, in turn, that a new entry for this user and every service output topic is added to the users_topics_map table.

Furthermore, our platform provides to users the possibility to start or stop the services for which they are owners, meaning that they have the ability to execute the source code file they have uploaded for this service on the platform’s infrastructure. Once the service is started (e.g., the source code file is executed as an operating system process or within another docker container), it subscribes to all IoT service input broker topics, it processes data received on these topics as instructed by the implemented algorithm, and it publishes the result (a stream of data) to its output topics. In order to manage the status of the service, there is an additional table in the database model services_process_info in which operating system-specific information (e.g., a process identifier) is stored in relation to the service “id”. This way, at any point of time, we can query whether the service is running to expect data on its output or whether its execution has stopped.

**Figure 2:**
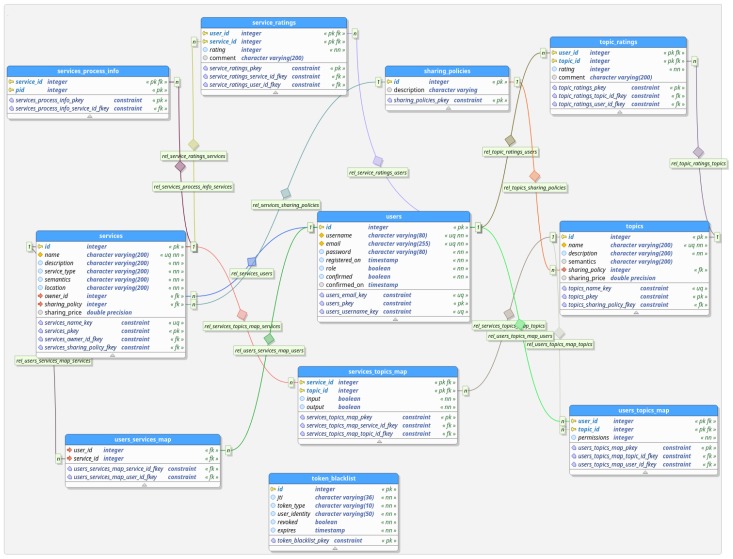
Database model.

Moreover, we have three additional tables that hold information regarding the ratings and the sharing policies. When a user submits a service or topic rating, then the corresponding data are inserted as a new row in the respective table. Finally, the table-sharing policies are used to provide predetermined sharing policy types for the user to choose among. The tables, fields, and relations that are defined in our data model are shown in Figure 2.

### 3.8. User-Access Control

When two clients wish to register for messages, they exchange SUB (subscribe) and PUB (publish) requests to the message broker. In fact, the subscribe requests are used by clients to subscribe to a topic, and as soon as another client publishes a message to that same topic, the message broker should determine whether these clients have adequate access rights in order to respectively send or receive these messages over that specific topic. In order to achieve a flexible and dynamic control over the user-access control process, our platform’s brokers are configured to use authentication extensions, which provide the possibility to send AuthN/AuthZ requests to third-party systems.

Thus, the SYNAISTHISI portal provides appropriate endpoints which receive as input user identities, user credentials, topics, and the desired actions, i.e., PUB/SUB, and which check against the information contained within the platform whether the specific user has the specific rights over the specified topic. If the requested actions are valid, an http 200 response is returned. If not, it responds with a deny (http 400) status code.

### 3.9. Service Management

In order to exploit the messages exchanged through the message brokers, to possibly perform some useful transformations on them, and thus to provide information that can be exploited by other clients, it is required that the IoT services that are running have subscribed to (input) topics and can publish to (output) topics. To explain further, recall that we implement IoT resource functionality as services. These services are programmed according to source code files and are attached (uploaded) to IoT services models created in the SYNAISTHISI portal. Our portal provides a service manager interface where users can upload respective files (connected to the IoT service they have created), can start or stop these services, and can also monitor their status (check their execution status and also download log files). As soon as the user chooses to start an IoT service, the portal executes a new process running the source code file, and as soon as this code file is enabled, it registers itself as (input) topic subscribers. Then, whenever it receives data on the subscribed topics, it implements a process based on this data and can publish back to its output topics, where some other clients may have subscribed to and, consequently, will receive the same data and provide useful interactions.

## 4. IoT Marketplace Approach

Apart from the technical requirements, to enable the formation of a large-scale IoT ecosystem, we need to provide adequate incentives to end users in order to keep them engaged [31]. Various types of incentives exist and can be incorporated, such as increased financial profits for active and effective contributors, user rankings and reviews, ease-of-use, expandability and reusability of existing solutions, etc. Such functionality should be offered in a form of an IoT marketplace that can be built on top of the technical platform that we described in the previous section.

First of all, in order to keep participation open, users should be able to freely create their accounts in the platform. To maintain control, administrative-type accounts should be able to manage user accounts and to possibly delete them, e.g., in case the terms of use are violated. Now, as previously discussed, to create IoT applications, a user should define the appropriate data streams or *topics* and the actual executable P-type services. Thus, each user is allowed to create, edit, and delete platform definitions regarding services and topics. Topics, services, and user account creation can be performed via the portal’s REST API or simply by using the GUI of the platform.

By default, only the creator of the resource is allowed to read or use data from topics and the source-code from these services. However, according to the user’s preference, the *sharing* of IoT resources is allowed by choosing between a number of sharing policies. Examples are *free access*, *no access*, *one-off fee*, and *pay-per-use*. No access can be used for private data, which a user would not agree to share, or even for services that contain proprietary source-code and where sharing is prohibited. When the free-access policy is selected, a user can request subscription rights to topics and the ability to redeploy existing services created by another user. In the one-off choice, the user is charged a predefined fee before access is granted. Finally, in the pay-per-use case, charging is performed according to the message data rates for topics and the CPU and memory usage that an executed service uses.

To promote service reusability, a user can request access to a service or topic that another user has created and owns. Respectively to the selected sharing policy, a user may request and be granted access to subscribe to other users’ topics or to deploy another user’s service. In order to be able to charge users for per-use access to data streams and services, the subscription requests and data transmission rates for each topic as well as the services execution and their computational resource use must be monitored and logged. This could also be performed as an inherent portal functionality with all relevant measurements being stored in the integrated database of the platform. Then, service and topic usages are charged to each account according to logged usage and the selected sharing policy. Apart from incentives of financial type that can be offered to contributors, social types may be employed as well. One kind of social incentive is to rate the available resources, where users can submit their levels of satisfaction regarding an IoT resource. Submitting comments in the form of plain text to include further explanations of the provided rating is also allowed.

Another feature that further simplifies platform usage by enabling effective IoT resource discoverability is to semantically annotate topics and services. To support this, the platform incorporates semantic descriptions of IoT resources, based on ontologies of specific standardizations. Specialized modules can be enriched with additional relevant ontologies according to the will of the platform administrator. At the first level, the user is able to select a semantic description to accompany the submitted IoT resource in order to be able to filter results based on this description later, as a means for IoT resource discoverability [53]. At later stages, semantic annotations can be also used for delivering automatic service composition techniques, where existing services are automatically combined, deployed, and evaluated to possibly generate better results [8].

Next, to enable quick development and deployment of complex IoT services, we seek to allow service reusability. To achieve that, we must make sure that the interconnection of existing services as well as the employment of multiple application layer protocols are also possible. By overcoming compatibility issues and implementation technology agnostics, the user can seamlessly interconnect services based merely on their functionality and logic, overlooking technical details such as supported platforms or programming language of choice. The inputs and outputs of a service are defined as abstract data streams, which, nevertheless, must be semantically annotated to match expected information flows.

The platform interoperability property, i.e., the ability to federate with different, possibly third-party, IoT platforms, is another important feature that a platform should integrate. Usually, this can be performed at various modes and levels, e.g., via gateways, by programmatically extending a platform or by developing specialized connectors, etc. Importantly, by being interoperable, the platform also extends the marketplace content, since services from other platforms can be offered as off-the-self solutions as well. For examples of platforms federation scenarios, see, e.g., the BigIoT [33] and symbIoTe [10] research projects. Finally, to realize a sustainable IoT services marketplace, active user participation is required. To this end, special communication channels must be established, either by mailing lists or by subscribing in source-code repositories. These channels can be used to distribute tutorials, advertisements, and success stories of IoT applications in real-world cases.

We now proceed to describe the design process for creating complex IoT services using the SYNAISTHISI platform, which can be incorporated in a multitude of use cases. We aim to illustrate how simple and heterogeneous IoT services can be reused, reconfigured, and unified to form more complex applications that can in, turn, be equipped by other users.

### 4.1. Designing IoT Services for AmI Use Cases

To help new users reduce the slope of the learning curve for developing platform-compatible IoT resources, the containerized platform version is shipped together with the source code of operable Sensing, Processing, and Actuation (SPA) services as well as with a generic service template that can be extended and used as the basis for the development of any type of IoT-enabled service that is hosted on SYNAISTHISI. More specifically, the generic service template offers a dummy functionality, which, nevertheless, illustrates the required library imports, input parameters, methods, and variable instantiations as well as the main structure that the source code of a given SPA service should have. The source code includes comments and indications to where the developer should focus on for programming the service-specific functionality, decreasing the time spent for understanding the basics and allowing for larger slopes of the learning curve required to begin using the SYNAISTHISI platform.

Moreover, additional operable SPA service examples used to showcase more advanced service capabilities can be included, such as sensing service reconfiguration and management, SPA service interconnections, and processing service executions that make use of well-known and specialized third-party libraries, e.g., the OpenCV library [54]. All available examples are based on the Python programming language; however, any other language can be used as well.

#### 4.1.1. Generic IoT Service Template

We continue by explaining the different parts of the generic service template that a user should customize/expand in order to build a SYNAISTHISI-compliant service. These parts are the header, the arguments parser, the definitions of callbacks, and the broker module initialization and instantiation.

##### Header

In this part of the generic service template, the developer sets the library imports that are being used in the source code. Here, apart from the required library imports (e.g., time, sys, argparse, and paho.mqtt.client), the developer can indicate any other Python library to be imported as well. This source code section can also include constant variable definitions, e.g., the internet protocol (IP) of the broker in case it is not set as an input argument and we want it to be “hardcoded” in our file.

##### Arguments Parsing

This section is required in order to pass the various configurable arguments to our service when it is executed. In order to be compliant with the SYNAISTHISI platform, each service should imperatively be accompanied with the following arguments:

- *username*: This argument passes a single parameter, the username of the user’s account as registered via the platform’s portal
- *password*: This argument passes a single parameter, the password of the user’s account as registered via the platform’s portal
- *input topics*: This argument is used to pass the input topics. The number of parameters given can be more than one.
- *output topics*: This argument is used to pass the output topics. The number of parameters given can be more than one.

Note that, accordingly, the end user can define any additional arguments, for example, the personalization parameters that will be used by each service, such as thresholds, measurement frequency, etc.

##### Callback Definitions

In this section, the callback functionality must be defined, i.e., what instructions should be executed when particular events happen with respect to connection status and message arrival. This way, we override the methods provided by the corresponding broker library and we can customize the operation of the developed service. Specifically, the callbacks that should be overridden are the following:

- *on connect ()*: This callback is immediately called when the connection with the platform’s broker is established. Apart from a possible informative message printed in the service’s output, this is the place for the user to initiate the subscriptions to the desired input topics. Note that, if the subscriptions are not initiated in the *on connect()* callback, then if the connection with the broker drops abruptly and then recovers again, the subscriptions need to be initiated again. If they are executed in this particular callback, however, this will be performed automatically.
- *on disconnect ()*: This callback is executed when the connection with the broker drops. Apart from printing informative messages in the service’s output, here is the place to put any additional instructions that the service should perform when unable to communicate with the IoT platform (e.g., transition to “no-operation states”, notification issuing, etc.).
- *on message ()*: This final and most important callback is called when a message arrives at one of the subscribed topics. Here, the user must define what needs to be performed for each input topic’s case.

##### Broker Library Module Initialization and Instantiation

In this part, the initialization of the required broker library methods and variables is performed. For the MQTT client cases, this category includes the software module that manages broker connections with a particular client “id” used by the message brokers, which can be also set to a random value. Following this initialization, we override the default client’s callbacks with the methods that we defined in the “Callback Definitions” section of the generic service template. Finally, the program must initiate the connection and engage in an infinite loop while waiting for incoming messages.

Having described the modules that must be implemented or overridden in order to develop a service of any type, we proceed to describe three IoT-enabled example use cases. These applications are composed of sensing (S)-, processing (P)-, and actuation (A)-type services, all of which can be based on the generic service template. The two of them are related to ambient intelligence applications, and one is related to industrial environments, among others.

#### 4.1.2. Face Counting on Video Streams

**Figure 3:**
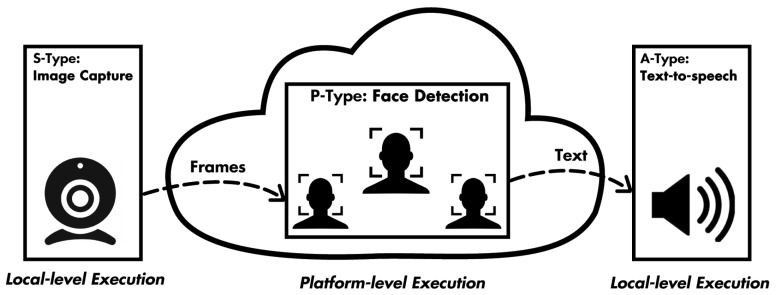
Composition of the face-counting service.

This service simply counts the human faces that are present in frames from video streams. The service uses three different micro-services: a web camera as an sensing-type service (S-type), either embedded on a computer or accessed via a USB (Universal Serial Bus) port; a processing-type service (P-type) that performs face detection on a static picture and publishes text with the number of human faces discovered; and an actuation-type service (A-type) that receives the number of faces in text format and performs a text-to-speech operation resulting in the announcement of the number of faces by a speaker. The schematic description of this complex SPA service is shown in Figure 3, and the related broker topics are given in Table 1.

As we can see, the S-type and A-type services run on a local level; however for the P-type, it is possible to be hosted by the IoT platform itself, and as a result, it can be executed either locally or on a cloud infrastructure.

#### 4.1.3. Fall Detection and Route Monitoring

In this scenario, we incorporate a number of mobile nodes that measure and post values from GPS and accelerometer sensors and a web-based dashboard that aggregates the measurements and can be used as means of monitoring and visualization. Such a service could be used in many use cases, for example by caretakers that supervise elderly people and patients in rehabilitation, kindergarten managers that need to know immediately if children are safe, etc. We note that, as a mobile node, readily available smartphones can be used as well. These S-type services constantly send measurements at frequent time intervals, e.g., 1 s, to specific topics that are registered on the platform by user accounts of the individuals that carry each device. Next, a P-type service instance, one for every mobile node, processes the measurements and (a) detects abnormalities, e.g., that a person falls according to the accelerometer measurements, and (b) translates GPS signals to map coordinates. On a final level, a web-based dashboard that subscribes to the output topics of the P-type services—after requesting permission and being granted by the individual personal accounts—aggregates and presents information in a visualized manner, e.g., red highlights for detected falls, green for normal measurements, map of premises with the respective geolocation data of the mobile nodes, etc.

**Figure 4:**
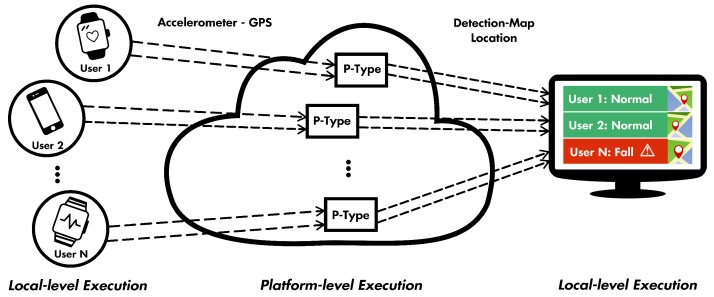
Composition of the fall-detection and route-monitoring service.

For this use case to work, the topics shown in Table 2 must be created independently for every user and appropriate access rights should be granted to the account of the aggregator. Next, the web-based dashboard subscribes to the output topics of each P-type service and processed measurements are effectively aggregated to be used by caretakers, managers, and so on. The micro-services used for this scenario are depicted in Figure 4.

From these two examples, it is apparent that the generic service template can be extended to and from services of any type and functionality. Furthermore, the platform’s support for various ALPs allows the interconnection of existing heterogeneous services that can be reused and reconfigured in complex application scenarios.

#### 4.1.4. Smart Factory Environment

In this part, we consider a general smart factory setting and discuss the way the proposed platform can be integrated. To begin, in the context of a smart factory, there exists modular manufacturing units equipped with intelligent controllers. Such units are able to receive reconfiguration commands, to communicate their status or measurements, to interconnect with other units or cloud services, and to be used to realize cyber-physical systems by collaborating with the factory personnel [55]. The data available in such settings have large volumes originating from multiple sources and can be manipulated by a number of processes, e.g., real-time monitoring, supply-chain analysis, product quality control, and so on. The protocols used for these processes are not necessarily supported by all the respective units; thus, protocol translation capabilities can come in handy.

Moreover, the nature of each process and operation has different requirements regarding, e.g., the ALP architecture that is incorporated and the responsiveness and latency in message exchange. For example, configuration and reconfiguration commands can be issued on-demand, thus requiring a request/response protocol such as HTTP/REST or CoAP. On the contrary, the exchange of messages originating from reactive components that should demonstrate a real-time operation require publish/subscribe protocols, so MQTT or AMQP would be more appropriate. Next, data aggregation modules could use both ALP architecture types; could publish/subscribe in cases where online measurement updates are imperative; and could request/response for data summarizations or sampling for various purposes, e.g., quality assessments, etc. In the same spirit, cloud and edge platform deployments are both required according to the nature of the application. In case some message topics are used for the abrupt operation of a production site, the platform should be deployed on a secure environment located inside the premises of the site. This way, issues in the external communications network will not halt production or lead to uncertain states of the equipment. Historical data and big data analytics modules, on the other hand, could be hosted on the cloud, allowing access to their resources from anywhere in the world, so that it can be shared with the industry’s executives and business analysts or even with third parties to create added value from this data.

Consider a smart industry setting where different applications or devices need to communicate data but they do not implement the same application layer protocols. For example, in a LoRaWAN (Long Range Wide Area Network), IoT devices send data to the network through LoRa gateways (https://lora-alliance.org). LoRa gateways forward data packets over Transmission Control Protocol/Internet Protocol (TCP/IP) to LoRa servers, which, in turn, process these packets and extract the useful payload. Such a LoRa server infrastructure is implemented by an open-source project, called LoRaServer. The components provided by the project offer the possibility to send uplink data to an endpoint over HTTP after having specified the preferred method (e.g., “POST”) and optionally having set the HTTP authorization headers. Now, in the case that an MQTT client (e.g., a Python processing application) would want to subscribe to this data and respond accordingly, by using the protocol translation capabilities of our platform’s brokers, an HTTP endpoint for the integration with the LoRaServer is offered and, upon receiving the data, this is published to the respective MQTT topic. That topic is where the client has subscribed to in order to receive the information. Otherwise, the client should continuously query the HTTP broker endpoint for updates, but it is quite more effective to subscribe to a topic and wait until the LoRaServer posts data to the HTTP endpoint.

### 4.2. Considerations for Real-World Deployments

Although the proposed approach adheres to available guidelines and specifications regarding applicability, horizontal scalability, etc., there are always limitations regarding the choices of how the platform is set up. First of all, performance is limited by the computational capabilities of the machines that host the platform. In cases that computationally intensive P-type services are required, these should be hosted on adequately strong servers or clusters of servers, since the frameworks that are used by the platform can all be executed in a distributed manner, e.g., by using message-broker bridges, database sharding techniques, and docker tools for server orchestration. Next, although the proposed solution supports a multitude of ALPs, proprietary protocols might be present in many sites, either domestic, commercial, or industrial, which are not directly supported. However, in most cases, special purpose connectors or translators exist that translate the proprietary protocol to one or more of the most widely adopted open ones.

Furthermore, there is always the bottleneck of network speed and available bandwidth that can significantly affect performance and quality of service of the IoT applications. An answer to this, apart from the incorporation of 5G networks, is the offer of flexible platform deployment capabilities, either on the cloud or on premises, therefore guaranteeing that a platform node will always be available regardless of the public network state on locations of interest. Finally, here, we do not define or suggest any specific data model or structure for the content of the exchanged messages, requiring an extra step by developers in the application design process. It is on the end users’ hands to choose the appropriate data types and structures that are the most suitable for each respective application.

## 5. Experimental Results

Having described the motivation, architecture, and implementation details, in this section, we perform stress tests to a locally deployed SYNAISTHISI container in order to identify the effective message rates and relative end-to-end message travel delay times. Importantly, we examine the impact that a protocol of choice might have with respect to the same performance indicators. First, we explain the simulation setting and define the control variables of the experiments. Then, we present the results from the simulations and draw important conclusions regarding which protocol should the designer choose to use and under which conditions.

One of our purposes in this section is to measure end-to-end message travel times in the presence of variable platform loads in the background. In particular, we have developed batch data publishing services, where the number of parallel publishers and subscribers can be configured, as well as the publishing frequency, the number of topics, and the message length in characters. Now, having these batch data publishers constantly exchanging messages at increasing numbers, we measure from another service the time delay between publishing a message and receiving it on a subscribed topic. This test is performed for the two supported publish/subscribe ALPs: MQTT and AMQP.

The other purpose of our experimentation is to compare end-to-end message delivery times when incorporating combinations of ALPs in the presence of batch data being sent and delivered using one ALP or the other. In this simulated setting, the number of parallel clients, the number of topics, the frequency, and the message length in bytes are all set to constant values throughout all experiments. Also, in this setting, we test both ALP categories: publish/subscribe (MQTT and AMQP) and request/response (CoAP and HTTP). Note that, due to the heterogeneity of resources and computational capabilities in an IoT ecosystem, it is difficult to provide a common testing ground and to perform objective benchmarking. Thus, the numerical results presented in this section should be interpreted in a more relative sense, extinguishing the denominator of the common underlying computational infrastructure used.

All experiments where conducted using an Intel(R) Core(TM) i5-4440 CPU at 3.10 GHz, 4 cores, 8-GB random access memory (RAM) machine, equipped with a solid-state drive for hosting the platform container. Services were executed on a separate machine, which, nevertheless, was connected under the same local area network (LAN), allowing speeds of up to 100 Mbps.

### Comparison of Protocols

**Figure 5:**
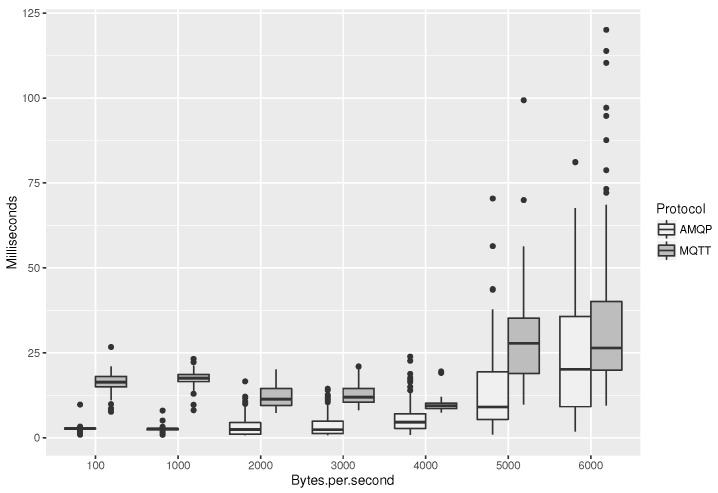
End-to-end message delays vs. batch message traffic size.

We begin by increasing the number of parallel batch publishing clients while using MQTT or AMQP as the protocol of choice. Results from the first experiment are shown in Figure 5. As we can see, the delay between message publish and receive as a subscriber is held relatively stable, below 25 ms for both protocols, in the first five iterations. In the rightmost two, the cases for 50 and 60 batch publishers exchanging messages at 5 and 6 KB/s, we have reached the physical limit of the platform hosting machine for normal operation and delays start to increase. Another observation is that AMQP has consistently lower end-to-end message delivery times than MQTT. This is due to the integrated implementation of AuthN/AuthZ into the RabbitMQ broker (handling the case of AMQP), as opposed to the case of Ponte, which incorporates the AuthN/AuthZ server of the Flask portal for the respective operations, introducing extra delays and complexity.

**Figure 6:**
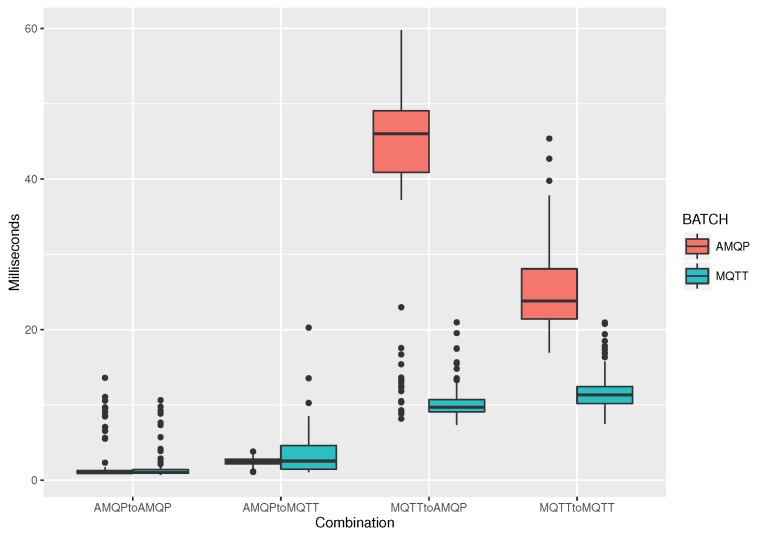
Parallel clients: 30, publish topics per client: 2, frequency of batch messaging: 1 s, batch message size: 50 B.

Next, we fix the number of clients to 30, each publishing on 2 topics every second with 50 Bytes of messages, and we examine the impact of ALP of choice in the end-to-end message travel times. Results are shown in Figure 6. As we can see, the faster performance is that of publishing and subscribing by using the AMQP protocol, regardless of the ALP used for batch publishing (leftmost couple of boxplots). Also, performance is similar when publishing in AMQP and when subscribing with the MQTT protocol, e.g., in a case of a cloud service issuing a command to notify remote actuators. However, we notice a significant increase in message delivery times when the publisher is using the MQTT protocol and when the subscriber is using the AMQP. In this case, the ALP used for batch data publishing also has impacts, since when AMQP is used, delays are multiplied by more than a factor of 2. In any case, 60 ms is found from our experimentation to be the upper bound for the publish/subscribe translations.

In Table 3, we show the average values from 10-s measurements regarding the use of the platform’s host machine resources. As we observe, there is a slight increase in RAM and CPU use when the AMQP protocol is used for batch publishing. Also, for the same cases (last four table rows), the size of inbound and outbound traffic also increased by a factor of less than 2. In general, as expected, the platform acts as a traffic multiplier, since incoming traffic volumes is multiplied by the number of subscribers.

**Figure 7:**
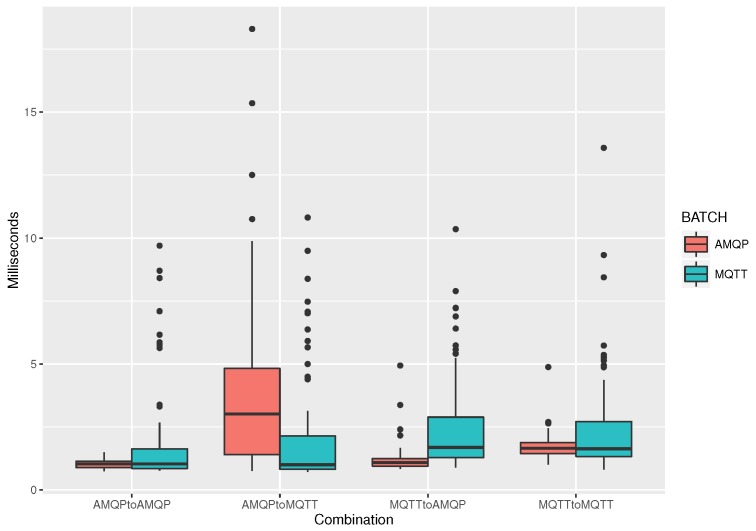
Without AuthN/AuthZ; parallel clients: 30, publish topics per client: 2, frequency of batch messaging: 1 s, batch message size: 50 B.

In a third experiment, to validate our argument for the extra delays in the MQTT case due to third-party AuthN-AuthZ server querying by the respective broker, we change the MQTT broker connection port of the client services to 1885, i.e., the RabbitMQ MQTT broker plugin, which uses the integrated AuthN/AuthZ server of RabbitMQ (which is, in turn, populated by the Flask portal). Results are shown in Figure 7. We can observe that, in this case, the performance of both publish/subscribe ALPs is quite similar, with the vast majority of messages being delivered within 10 ms after their publishing. For the case of MQTT batch messaging protocol of choice, it seems that data samples have more variance than in the AMQP batch data case; however, this does not hold when publishing from AMQP and subscribing using MQTT (second couple of boxplots).

**Figure 8:**
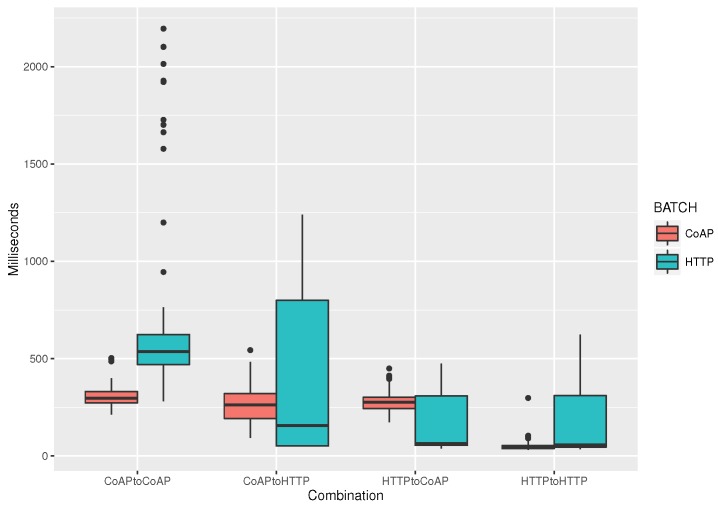
Parallel clients: 30, publish topics per client: 2, frequency of batch messaging: 1 s, batch message size: 50 B.

In a final simulation experiment, we test in a similar manner the translations between the two request/response ALPs: CoAP and HTTP. Results are shown in Figure 8. As we observe, both the average delay and the variance are of orders of ten higher than in the case of the publish/subscribe ALPs. This happens because the asynchronous nature of the request/response method implementation adds significant overhead between the time a message is sent to the broker and received by the client with a “GET” request.

Comparing the performance of the message translation between the two request/response ALPs, we see that it is faster to both send and receive messages using the HTTP protocol (last couple of boxplots) while having CoAP batch messages being sent in the background. In the other cases, HTTP batch data induced increased variance in the delays, while CoAP batch data publishing illustrated a more stable performance.

In Table 4, we present the 10-s measurements of the platform’s host machine resource use during the experiment with the request/response ALPs. As we can see, comparing also with Table 3, ALPs of this kind are quite more demanding in terms of RAM and CPU but generate quite lower network traffic. Differences between CoAP and HTTP batch message publishing are not found to be significant.

## 6. Conclusions and Future Work

In this paper, we analyzed an IoT platform that uses open-source middleware frameworks and integrates them into single, operable, dockerized instances. To deliver this, we combined open-source message-brokering frameworks that support and cross-translate the MQTT, REST/HTTP, Websockets, CoAP, and AMQP application layer protocols, as well as the oneM2M service layer specification. Moreover, to further promote interoperability, platform sub-modules are interconnected with an RDF triplestore for storing and processing semantic data that constitute the ontological characterizations of IoT resources. Also, the investigated platform supports authentication and authorization procedures regarding access and ownership of data streams and cloud-based micro-services, as well as means for their execution monitoring and management. Furthermore, we illustrated examples for the development of IoT-enabled complex services that can be reused in a variety of application areas. Multiple protocol support has been experimentally evaluated with respect to platform performance when different ALPs are used. The proposed approach can be used to promote the unification of fragmented IoT solutions, effectively managing to integrate verticals and to generate added value from services that use big data.

In the future, we plan to investigate additional strategies for creating revenue streams for the end users and to develop IoT services that effectively incorporate platform federation capabilities. This can result in complex applications that are composed of micro-services, possibly offered by third-party platforms. Therefore, large-scale and interoperable IoT ecosystems will be enabled, allowing major improvements to the operations of business and industry. Also, we plan on integrating additional communication protocols, such as Kafka and DDS (Data Distribution Service), and on offering more customizable and configurable graphical user interfaces. Moreover, it is of our interest to research methodologies for ontology evolution and expandability that will be used for inference and reasoning in automatic service composition processes [56]. Finally, we will focus on the development of generic services that incorporate Artificial and Ambient Intelligence techniques to be offered for free to platform users as default application examples.

## Figures and Tables

**Table 1:** Topics used by the face-counting service.

Topic	Description
Frame topic	Used to transfer frames from the sensing (S)-type to the processing (P)-type
Text topic	Used by the P-type service to notify the actuation (A)-type with updates

**Table 2:** Topics utilized by the fall-detection and route-monitoring service.

Topic	Description
User1 Accelerometer	Used to transfer accelerometer data of User1 from the S-type to the P-type service
User1 GPS	Used to transfer accelerometer data of User1 from the S-type to the P-type service
User1 Detection Result	Used to publish recognized abnormalities to the web-based GUI
User1 Map Location	Used to publish User1’s location to the web-based GUI
User2 Accelerometer	Used to transfer accelerometer data of User2 from the S-type to the P-type service
User2 GPS	Used to transfer accelerometer data of User2 from the S-type to the P-type service
User2 Detection Result	Used to publish recognized abnormalities to the web-based GUI
User2 Map Location	Used to publish User2’s location to the web-based GUI
...	...
UserN Accelerometer	Used to transfer accelerometer data of UserN from the S-type to the P-type service
UserN GPS	Used to transfer accelerometer data of UserN from the S-type to the P-type service
UserN Detection Result	Used to publish recognized abnormalities to the web-based GUI
UserN Map Location	Used to publish UserN’s location to the web-based GUI

**Table 3:** Use of server resources during each publish/subscribe experiment (Average 10-s values).

Publisher-Subscriber (Batch)	Free Memory %	CPU %	KB Received	KB Sent
MQTT-MQTT (MQTT)	70.79	21.52	840.97	2704.32
AMQP-MQTT (MQTT)	70.30	23.59	839.00	2703.45
MQTT-AMQP (MQTT)	72.31	23.23	842.71	2705.45
AMQP-AMQP (MQTT)	71.85	22.99	810.63	2703.08
MQTT-MQTT (AMQP)	67.98	26.79	1560.06	4054.54
AMQP-MQTT (AMQP)	67.61	24.92	1564.03	4071.11
MQTT-AMQP (AMQP)	67.66	26.42	1558.51	4140.26
AMQP-AMQP (AMQP)	67.15	26.19	1554.52	4140.10

**Table 4:** Use of server resources during each request/response experiment (Average 10-s values).

Publisher-Subscriber (Batch)	Free Memory %	CPU %	KB Received	KB Sent
CoAP-CoAP (CoAP)	35.46	79.47	26.28	35.14
CoAP-HTTP (CoAP)	33.94	84.24	27.93	36.19
HTTP-CoAP (CoAP)	33.83	84.29	26.95	35.48
HTTP-HTTP (CoAP)	34.52	84.94	29.32	36.78
CoAP-CoAP (HTTP)	35.94	84.81	28.91	36.41
CoAP-HTTP (HTTP)	34.83	84.47	27.81	35.99
HTTP-CoAP (HTTP)	33.50	84.70	28.52	36.94
HTTP-HTTP (HTTP)	33.47	86.46	29.96	37.92

## Abbreviations

The following abbreviations are used in this manuscript:

ALP	Application Layer Protocol
AmI	Ambient Intelligence
AMQP	Advanced Message Queuing Protocol
API	Application Programming Interface
AuthN/AuthZ	Authentication/Authorisation
CoAP	Constrained Application Protocol
CPU	Central Processing Unit
GPS	Global Positioning System
GUI	Graphical User Interface
HTTP	HyperText Transfer Protocol
IoT	Internet of Things
LAN	Local Area Network
MQTT	Message Queuing Telemetry Transport
RAM	Random Access Memory
RDF	Resource Description Framework
REST	Representational State Transfer
SPA	Sensing, Processing, Actuation
STOMP	Streaming Text Oriented Messaging Protocol
TCP/IP	Transmission Control Protocol/Internet Protocol
XML	Extensible Markup Language
